# Draft Genome Sequences of Three Strains of Verticillium nonalfalfae Exhibiting Different Levels of Aggressiveness on Ailanthus altissima

**DOI:** 10.1128/MRA.01384-19

**Published:** 2020-01-09

**Authors:** Harald Berger, Oliver Maschek, Erhard Halmschlager

**Affiliations:** a Institute of Applied Genetics and Cell Biology (IAGZ), Department of Applied Genetics and Cell Biology, University of Natural Resources and Life Sciences, Vienna, Austria; b Institute of Forest Entomology, Forest Pathology and Forest Protection (IFFF), Department of Forest and Soil Sciences, University of Natural Resources and Life Sciences, Vienna, Austria; University of Maryland School of Medicine

## Abstract

Verticillium nonalfalfae, a soilborne vascular fungus, shows promise for biocontrol of highly invasive Ailanthus altissima strains. This announcement provides draft genome sequences of the aggressive isolate G1/5 (wild-type strain), the highly aggressive isolate Vert56 (improved strain), and the mildly aggressive isolate I3/2, all obtained from symptomatic *A. altissima* trees in Austria.

## ANNOUNCEMENT

Verticillium nonalfalfae is an important vascular wilt pathogen on several dicotyledonous crops as well as on tree of heaven (Ailanthus altissima) (1–3). This phytopathogenic fungus seems to be a promising candidate for the biological control of this highly invasive tree species (4–7). For this reason, *V. nonalfalfae* isolate G1/5, originally obtained from a dying *Ailanthus* sp. in Styria, Austria, has been used in several consecutive inoculation experiments since 2011, forcing host adaptation and increased disease progression by selection (8) and resulting in the improved strain Vert56.

To support the hypothesis of host adaptation, comparative genomics of the wild-type strain G1/5 (GenBank accession numbers KT223526 and WGLJ00000000) (3), the improved strain Vert56 (GenBank accession number WGLK00000000), and another less pathogenic isolate, I3/2 (GenBank accession numbers KT223527 and SPUV00000000) (3), was carried out.

Fungi were grown on 1% malt extract agar, and genomic DNA was prepared from mycelium using the DNeasy plant minikit (Qiagen) with Milli-Q water for elution. Whole-genome sequencing was performed by Vienna BioCenter Core Facilities GmbH (Vienna, Austria) using an Illumina HiSeq 2500 system with a single-end 100-bp setup. Sequencing libraries were prepared according to the Westburg next-generation sequencing (NGS) DNA library prep kit protocol v3.1 (Westburg, the Netherlands). A total of 60.09 million (Vert56), 54.24 million (I3/2), and 57.87 million (G1/5) reads were received, representing in total 6,008.9 million, 5,423.5 million, and 5,786.7 million bases, respectively. Default parameters were used for all software, unless otherwise noted. A read quality check was performed using FastQC (v0.11.4) (9). Raw sequence reads were quality filtered using Trimmomatic (v0.36) (10) to finally obtain 51.6 million (Vert56), 50.2 million (I3/2), and 56.54 million (G1/5) reads that were used for assembly. The de Bruijn graph-based assembler SPAdes (v3.12.0) (11) was used for the scaffold assembly after estimating the optimal k-mer lengths using KmerGenie (v1.7023) (12). We obtained 901 (Vert56), 1,424 (I3/2), and 781 (G1/5) contigs longer than 1 kb. These contigs were connected by using another published *V. nonalfalfae* strain (2) as a reference to obtain 373 (Vert56), 273 (I3/2), and 345 (G1/5) supercontigs, with mean coverages of 153, 152, and 168 and total lengths of 33.6, 32.8, and 33.7 Mb, respectively. The quality of the genome assembly was assessed in QUAST (v5.0.2) (13), and the results are as follows: for Vert56, *N*_50_, 322,948 bp; *N*_75_, 178,814 bp; *L*_50_, 32; *L*_75_, 69; and G+C content, 55.15%; for I3/2, *N*_50_, 291,550 bp; *N*_75_, 179,670 bp; *L*_50_, 32; *L*_75_, 68; and G+C content, 55.26%; and for G1/5, *N*_50_, 300,109 bp; *N*_75_, 175,936 bp; *L*_50_, 32; *L*_75_, 68; and G+C content, 55.11%.

Sequencing completeness was estimated using BUSCO (v3.0.2) (14) based on a set of 3,725 sordariomycetic genes also known as Benchmarking Universal Single-Copy Orthologs (BUSCOs). A total of 3,652, 3,622, and 3,650 complete single-copy BUSCOs; 27, 29, and 28 fragmented BUSCOs; and 8, 7, and 7 duplicated BUSCOs were found, leading to 38, 67, and 40 missing BUSCOs in *V. nonalfalfae* strains Vert56, I3/2, and G1/5, respectively.

### Data availability.

These whole-genome shotgun projects have been deposited at DDBJ/EMBL/GenBank under the accession numbers WGLJ00000000 (*Verticillium nonalfalfae* G1/5), SPUV00000000 (*Verticillium nonalfalfae* I3/2), and WGLK00000000 (*Verticillium nonalfalfae* Vert56). The versions described in this paper are the first versions, WGLJ01000000, SPUV0100000000, and WGLK01000000, respectively. Sequence reads were deposited under SRA project accession numbers SRR8271219, SRR8271218, and SRR8271217, respectively, and BioProject accession number PRJNA507541.
